# Supplementary material for: Human running performance from real-world big data
Source: Nat Commun. 2020 Oct 6;11:4936. doi: 10.1038/s41467-020-18737-6 (PMC7538888; doi:10.1038/s41467-020-18737-6)
Supplement: Supplementary file 1 — Reporting Summary [file 41467_2020_18737_MOESM1_ESM.pdf]

nature research

Corresponding author(s): Thorsten Emig

Last updated by author(s): Aug 3, 2020

# Reporting Summary

Nature Research wishes to improve the reproducibility of the work that we publish. This form provides structure for consistency and transparency in reporting. For further information on Nature Research policies, see our Editorial Policies and the Editorial Policy Checklist.

## Statistics

For all statistical analyses, confirm that the following items are present in the figure legend, table legend, main text, or Methods section.

| n/a | Confirmed | |
|---|---|---|
| ☐ | ☒ | The exact sample size (*n*) for each experimental group/condition, given as a discrete number and unit of measurement |
| ☐ | ☒ | A statement on whether measurements were taken from distinct samples or whether the same sample was measured repeatedly |
| ☒ | ☐ | The statistical test(s) used AND whether they are one- or two-sided<br>*Only common tests should be described solely by name; describe more complex techniques in the Methods section.* |
| ☒ | ☐ | A description of all covariates tested |
| ☒ | ☐ | A description of any assumptions or corrections, such as tests of normality and adjustment for multiple comparisons |
| ☐ | ☑ | A full description of the statistical parameters including central tendency (e.g. means) or other basic estimates (e.g. regression coefficient) AND variation (e.g. standard deviation) or associated estimates of uncertainty (e.g. confidence intervals) |
| ☒ | ☐ | For null hypothesis testing, the test statistic (e.g. *F*, *t*, *r*) with confidence intervals, effect sizes, degrees of freedom and *P* value noted<br>*Give P values as exact values whenever suitable.* |
| ☒ | ☐ | For Bayesian analysis, information on the choice of priors and Markov chain Monte Carlo settings |
| ☒ | ☐ | For hierarchical and complex designs, identification of the appropriate level for tests and full reporting of outcomes |
| ☒ | ☐ | Estimates of effect sizes (e.g. Cohen's *d*, Pearson's *r*), indicating how they were calculated |

*Our web collection on statistics for biologists contains articles on many of the points above.*

## oftware and code

Policy information about availability of computer code

| | |
|---|---|
| Data collection | Data were collected by Polar Oy via their web service PolarFlow. |
| Data analysis | RStudio script Code is deposited in GitHub, see manuscript section "Code availability" for DOI. |

For manuscripts utilizing custom algorithms or software that are central to the research but not yet described in published literature, software must be made available to editors and reviewers. We strongly encourage code deposition in a community repository (e.g. GitHub). See the Nature Research guidelines for submitting code & software for further information.

## Data

Policy information about availability of data

All manuscripts must include a data availability statement. This statement should provide the following information, where applicable:
- Accession codes, unique identifiers, or web links for publicly available datasets
- A list of figures that have associated raw data
- A description of any restrictions on data availability

The data that support the findings of this study are available from Polar Electro Oy, Professorintie 5, 90440, Kempele, Finland, upon request.

# Field-specific reporting

# Life sciences study design

All studies must disclose on these points even when the disclosure is negative.

Sample size was determined by available data during period Jul 2015 to Dec 2018 provided by Polar. This size is by far sufficient, as indicated by smooth distribution functions.

| | |
|---|---|
| [illegible]ample size | Sample sizes were obtain from PolarFlow data by counting subject IDs and their activities. |
| [illegible]ta exclusions | Unreasonable data (too large velocities, too few training activities) were excluded, see Methods sections for details. Data exclusion criteria were pre-established. Data exclusion was applied to all available data from Polar Flow website. |
| Replication | no replication since one-time activity redcordings were used. |
| Randomization | no devisions of subjects into groups as our mathematical model was applied to all subjects in the same way. |
| [illegible]nding | blinding not relevant to our comparison of activity data from running and a mathematical model. |

Blinding was not relevant as this is not a clinical research study and differential assessment or treatment cannot occur since individuals uploaded their data to webservice before our study started, without knowing about our analysis. We did not use any personal information of individual besides their sex. All individuals and their activities were linked only by an anonymous ID.

# Reporting for specific materials, systems and methods

We require information from authors about some types of materials, experimental systems and methods used in many studies. Here, indicate whether each material, system or method listed is relevant to your study. If you are not sure if a list item applies to your research, read the appropriate section before selecting a response.

## Materials & experimental systems

| n/a | Involved in the study |
|---|---|
| ☒ | ☐ Antibodies |
| ☒ | ☐ Eukaryotic cell lines |
| ☒ | ☐ Palaeontology and archaeology |
| ☒ | ☐ Animals and other organisms |
| ☒ | ☐ Human research participants |
| ☒ | ☐ Clinical data |
| ☒ | ☐ Dual use research of concern |

## Methods

| n/a | Involved in the study |
|---|---|
| ☒ | ☐ ChIP-seq |
| ☒ | ☐ Flow cytometry |
| ☒ | ☐ MRI-based neuroimaging |
